# Making sense of transformer success

**DOI:** 10.3389/frai.2025.1509338

**Published:** 2025-04-01

**Authors:** Nicola Angius, Pietro Perconti, Alessio Plebe, Alessandro Acciai

**Affiliations:** Department of Cognitive Science, University of Messina, Messina, Italy

**Keywords:** philosophy of AI, philosophy of cognitive science, neural language models, deep learning, functional explanations, mechanistic explanations, simulative AI

## Abstract

This article provides an epistemological analysis of current attempts of explaining how the relatively simple algorithmic components of neural language models (NLMs) provide them with genuine linguistic competence. After introducing the Transformer architecture, at the basis of most of current NLMs, the paper firstly emphasizes how the central question in the philosophy of AI has been shifted from “can machines think?”, as originally put by Alan Turing, to “how *can* machines think?”, pointing to an explanatory gap for NLMs. Subsequently, existing explanatory strategies for the functioning of NLMs are analyzed to argue that they, however debated, do not differ from the explanatory strategies used in cognitive science to explain intelligent behaviors of humans. In particular, available experimental studies turned to test the *theory of mind, discourse entity tracking*, and *property induction* in NLMs are examined under the light of the *functional analysis* in the philosophy of cognitive science; the so-called *copying algorithm* and the *induction head* phenomenon of a Transformer are shown to provide a *mechanist* explanation of *in-context learning*; finally, current pioneering attempts to use NLMs to predict brain activation patterns when processing language are here shown to involve what we call a *co-simulation*, in which a NLM and the brain are used to simulate and understand each other.

## 1 Introduction

Roughly speaking, two main paths can be identified along which the rise of artificial intelligence has unfolded in the last ten years, driven by the new Artificial Neural Networks (ANN) and marked by Deep Learning (DL) (LeCun et al., 2015; Goodfellow et al., 2016). In the first five years, the most successful path was vision, leading for the first time to artificial systems with a visual recognition ability similar to that of humans (Krizhevsky et al., 2012; Simonyan and Zisserman, 2015; Szegedy et al., 2015) arousing surprise and interest in the science of vision (Gauthier and Tarr, 2016; VanRullen, 2017; Grill-Spector et al., 2018). Five years later, it was the turn of language, a path opened by the Transformer model (Vaswani et al., 2017), quickly followed by various evolutions and variants (Devlin et al., 2019; Brown et al., 2020; Ouyang et al., 2022; Touvron et al., 2023), generically called here Neural Language Models (NLMs). In this case too, the sudden and unexpected availability of artificial systems with linguistic performances not so far from human ones has deeply shaken the scientific community of language scholars (Alishahi et al., 2019; Baroni, 2019; Boleda, 2020; Green and Michel, 2022; Pavlick, 2023). But the stakes are much higher along the path of language than in that of vision. Even though seeing is a fundamental ability of human beings, it is marginally linked to the common account of intelligence. Understanding, and being able to express oneself correctly in a language, on the other hand, goes hand in hand with being–at least to some extent–intelligent, being able to think and reason.

Understandably, the appearance of the NLMs has revived the ancient debate about whether or not a machine can think, be intelligent (Bender and Koller, 2020; Rees, 2022; Søgaard, 2022; Agüera y Arcas, 2022; Floridi, 2023; Perconti and Plebe, 2023; Søgaard, 2023). This article starts by arguing that the question of whether machines can think (Turing, 1950), is not to be taken any more as the central one in the philosophy of Artificial Intelligence (AI). The crucial philosophical issue has become that of providing explanations for this ability. This does not imply that the matter has been settled, at least not in the sense that all philosophical doubts concerning the very possibility of artificial semantics have been dispelled. Nevertheless, the fact that some NLM performances now appear genuinely intelligent suggests that we should also examine how this phenomenon may have arisen. It should be emphasized that the explanatory request under focus here does not concern the algorithmic components of Transformer–based models, on which there is plenty of technical descriptions. The question addressed in this article is rather how the relatively simple algorithmic components of the Transformer provide it with the ability to produce linguistically adequate outputs and to reason at a level comparable to humans. It's worth noting that while linguistics has generated highly sophisticated and detailed descriptions of language, how it is understood and generated by a brain remains essentially a mystery, much like in NLMs. One of the ambitions of AI has been to explain aspects of natural cognition by designing their equivalents. However, the presupposition was that these artificial equivalents would be understandable, which is not the case with NLMs. It's challenging to determine whether shedding light on how NLMs function can contribute to understanding language in the brain.

The almost total absence of explanations for the linguistic abilities of the NLMs contrasts with the relative simplicity of their computational architecture and their way of learning. Again, there is a vast technical literature that computationally illustrates the implementations of the various NLMs (Tingiris, 2022; Rothman, 2022), but there is a huge gap from here to identifying what in these implementations gives language faculty. One of the best illustrative texts on Transformer architectures (Wolfram, 2023, p. 71) underscores the issue well: “It has to be emphasized again that there's no ultimate theoretical reason why anything like this should work. And in fact, as we'll discuss, I think we have to view this as a—potentially surprising—scientific discovery: that somehow in a neural net like ChatGPT's it's possible to capture the essence of what human brains manage to do in generating language.”

The line of reasoning proposed here might open up to a fundamental objection.^1^ To be legitimately allowed to shift the discussion from *can* a machine think to *how* machines can think, it would seem indispensable to have first ascertained that in a philosophically proper sense machines *can* think. We call this objection *can comes first*. It seems appropriate, given that the question of whether the performances of NLM can qualify as *thinking* is vigorously debated in philosophical terms. The *can comes first* objection will be addressed in detail in §3, where it is shown how it can be overcome by distinguishing between possibilities in a metaphysical sense and in an empirical sense. We pursue the latter, which legitimizes the transition to the new problem of *how* machines can think.

The explanatory request arising from NLMs is in this paper addressed by providing an epistemological examination of the current literature analysing the performance of NLMs in many intelligent tasks. In order to do so, Section 2 firstly introduces the Transformer architecture. Subsequently, Section 3 brings up for NLMs the old question of whether machines can think, addresses the *can comes first* objection, which directly leads to the question that we currently consider more pressing, namely *how* machines *can* think. Then, three sections epistemologically analyse different directions taken by the early existing attempt of explanations in terms of functional explanations (Section 4), mechanistic explanations (Section 5), and simulative explanations (Section 6). Finally, Section 7 concludes the paper by emphasizing, one the one hand, how the explanatory strategies advanced to explain NLMs do not differ from those used in the philosophy of mind to explain the behaviors of natural cognitive systems; on the other hand, how the explanatory gap for NLMs challenges the simulative, or synthetic, method in cognitive science according to which simulative systems are used to understand the mind.

## 2 Neural models and natural language

The first attempt to incorporate Artificial Neural Networks (ANNs) into the field of natural language processing was made by Rumelhart and McClelland (1986), with a focus on inflectional morphology. Their model was successful, however, they encountered a significant challenge in using artificial neural models for language processing. Language is an ordered sequence of symbols, while a neural layer is a real vector with a fixed dimension. This makes it problematic to encode an arbitrary length datum with a vector of fixed dimensions even for models confined to the processing of single words.

A second difficulty is that representing words with neural vectors worsens when transitioning from single-word morphology to syntax. Feedforward ANNs are static, and establishing a sense of ordering for multiple words in a sentence is far from straightforward. Elman (1990) proposed an elegant solution by supplementing the hidden layer of a feedforward network with a so-called context layer, featuring recurrent connections between the hidden layers. However, severe limitations arise as soon as one moves from demonstrations on simple short sentences to full language processing: recurrent networks struggle to maintain relevance for words that are too distantly placed, yet syntactically related. An additional difficulty for traditional ANN stems from the very technique that had decreed its success in the '90s: backpropagation learning (Rumelhart et al., 1986). Its efficiency comes at a price: supervision, where the correct outputs for input samples are known. The ability to understand language, and even more so to produce it, goes beyond tasks where the necessary inputs and outputs for supervised training can be clearly identified.

The Transformer architecture, invented by Vaswani et al. (2017) at Google, combines several effective strategies that address all the three difficulties. First is the *neural word embedding* technique, which learns from examples the optimal way to transform words into vectors of neural activity. The first neural word embeddings were introduced by Bengio et al. (2003), and recently significantly improved by Mikolov et al. (2013). Their primary feature is that the vectorial representations seem to carry information that corresponds to meaningful distinctions for humans.

The numerical vectors can be manipulated, yielding results that interestingly respect aspects of lexical semantics. Let $\vec{w}\left(\cdot \right)$ be the word embedding transformation, by computing:


$$\begin{array}{l} \vec{q}=\vec{w}\left(king\right)-\vec{w}\left(male\right)+\vec{w}\left(female\right) \end{array}$$

The resulting vector $\vec{q}$ is more similar to $\vec{w}\left(queen\right)$ than to any other word embedded vector. The second strategy is the attention mechanism (Bahdanau et al., 2016). This technique dynamically identifies relevant information and relationships among words in a sentence. The Transformer employs these strategies innovatively. First of all, word embedding is learned while the NLM captures contextual patterns, semantic relationships and syntactic regularities from the corpora. Secondly, the attention mechanism entirely replaces recursion. Now, all words, along with their vectorial embedding, are simultaneously presented as input. Furthermore, the Transformer adopts an elegant solution that allows us to bypass supervised learning (Hinton and Zemel, 1994): the concept of the *autoencoder*. This disarmingly simple idea is that the task assigned to the ANN is to reproduce its own input. The architecture that implements it is typically organized into two components. The encoder is responsible for producing an internal representation of the input, and the decoder reproduces the output from this representation, which coincides with the input. It should be emphasized that the learning strategy of an autoencoder conceals what a NLM actually learns from the data it is exposed to. In other DL systems, such as visual classifiers, the loss through which synaptic weights are adjusted is explicitly related to their task. Taking, for example, a DL vision system used in a self-driving vehicle, its final layer will represent categories such as cyclists and pedestrians. During training, the loss will be due to misclassification, for example, mistaking a pedestrian for a cyclist. This loss precisely clarifies what the system is expected to learn, which is its anticipated task: to indicate that there's a pedestrian in the scene when it is indeed a pedestrian, and similarly for a cyclist. In NLM, however, the error in predicting the next word in the sentence is irrelevant compared to what the model captures of the expressiveness of natural language from this simple loss. Chalmers (2023) has emphasized the irrelevance of loss in relation to what the models truly learn with this effective metaphor:

“in evolution by natural selection, maximizing fitness during evolution can lead to wholly novel processes post-evolution. A critic might say, all these systems are doing is maximizing fitness. But it turns out that the best way for organisms to maximize fitness is to have these amazing capacities—like seeing and flying and even having world-models.”

**Figure 1:**
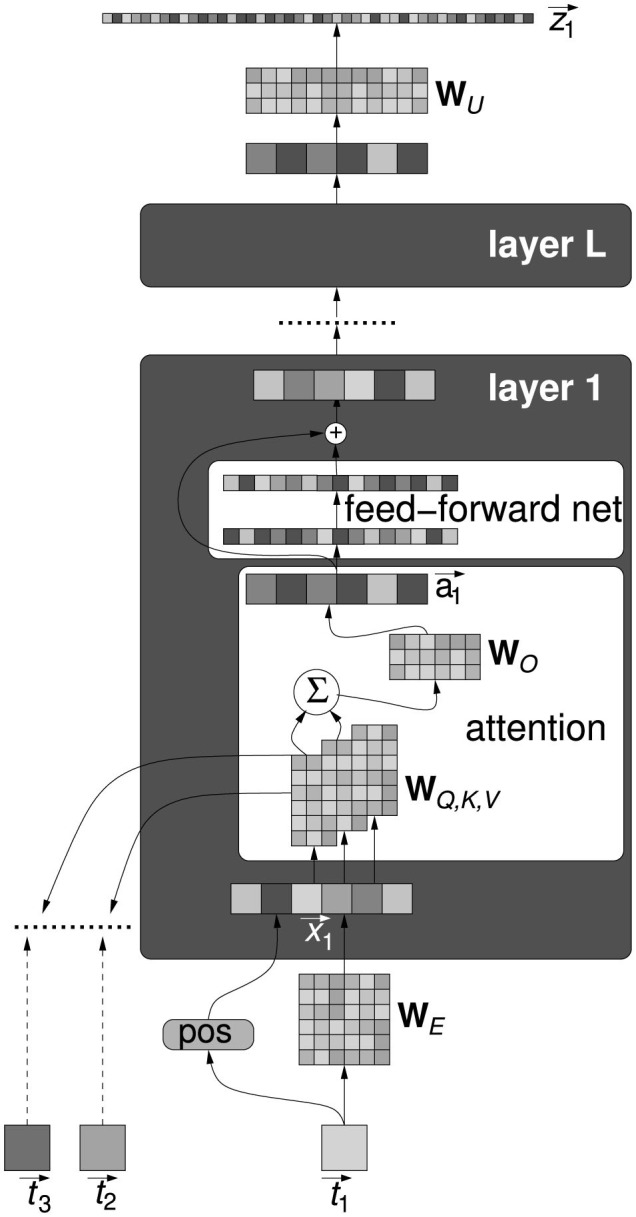
A simplified scheme of the overall Transformer architecture. All components are described in the text.

What kind of world-models have emerged in a NLM following training is part of the endeavor to make sense of how the Transformer works. The unexpected remarkable efficiency of the Transformer has triggered its substantial development, gradually untethered from the original narrow aims of automatic translation, toward dialogue and autonomous text generation (Devlin et al., 2019; Brown et al., 2020; Ouyang et al., 2022). Herein, a simplified description of the Transformer is provided, useful within the scope of this article for the subsequent presentation of some attempts at a mechanistic explanation of its capabilities, an overall scheme is shown in Figure 1. The input text is made by *tokens* *t*_*i*_, where each token is an integer index into the vocabulary, made by words together with punctuation marks and also parts of words. The size of the vocabulary *N* is typically of several tens of thousands. A crucial operation on the input token is embedding, performed with the embedding matrix ${\text{W}}_{E}\in {\mathbb{R}}^{D\times N}$, with *D* the embedding dimension. For a token *t*_*i*_ in the input stream the embedded vector is computed as follows:


(1) $$\begin{array}{l} {\vec{x}}_{i}={\text{W}}_{E}^{\left(t_{i}\right)}+p\left(i\right) \end{array}$$

where ${\text{W}}_{E}^{\left(j\right)}$ is the *j*-th column of W_*E*_ and *p*(·):ℕ → ℝ^*d*^ is a function that encode the position of the token inside the stream of text.

The model consists of a chain of *L* layers, in each layer an attention block is followed by a feedforward neural network, each block reads from and writes to the same residual stream. Figure 1 displays in detail only one layer and for one token only, all tokens are processed in parallel. The output of the last layer is mapped back to the vocabulary space by the unembedding matrix ${\text{W}}_{U}\in {\mathbb{R}}^{N\times D}$ and then fed into a softmax layer. Each element in the output vector ${\vec{z}}_{i}$ represent the probability of a token to be the successor of ${\vec{t}}_{i}$.

**Figure 2:**
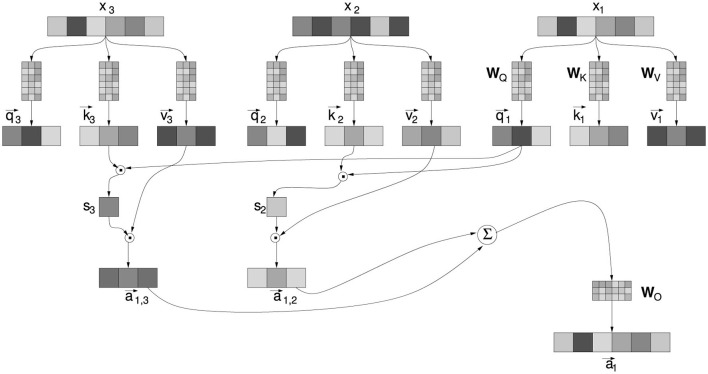
Detail of the attention mechanism, for the current embedded token ${\vec{x}}_{1}$ with respect to the previous tokens ${\vec{x}}_{2}$ and ${\vec{x}}_{3}$. All elements are described in the text.

A zoom into the attention mechanism is provided in Figure 2. It is based on linear algebra operations using the following matrices:

- ${\text{W}}_{K}\in {\mathbb{R}}^{A\times D}$ – the “key” matrix;
- ${\text{W}}_{Q}\in {\mathbb{R}}^{A\times D}$ – the “query” matrix;
- ${\text{W}}_{V}\in {\mathbb{R}}^{A\times D}$ – the “value” matrix;
- ${\text{W}}_{O}\in {\mathbb{R}}^{D\times A}$ – the “output” matrix.

*A* is the dimension of the vector used in the attention computation, in most current NLMs is equal to *D*. The matrices W_*K,Q,V*_ map an embedded token into the vectors “query” $\vec{q}$; “key” $\vec{k}$; and “value” $\vec{v}$. The scalars *s*_*i*_ in Figure 2, called “score”, result from the multiplication of the “query” and “key” vectors, and modulate the amount of the “value” vectors. The terms “key”, “query”, and “value” are remnants of the common jargon in information retrieval (Salton and McGill, 1983) and associative memory systems (Anderson and Bower, 1974), where “query” expresses what one is searching for, “key” is the index that best matches the “query”, pointing to a “value” which is the sought-after content. However, it should be noted that in the Transformer, these interpretations should not be taken too literally, just as “attention” should not be taken as a synonym for the psychological mechanism bearing the same name. Indeed, in the Transformer, there is no predefined index to compare with an explicit query and retrieve content. All these vectors are linear transformations of embedding vectors through the W_*K,Q,V*_ matrices. Just as it happens for the weights of the feed-forward components, all elements of these matrices are learned through the minimization of the same loss: the prediction of upcoming tokens in sentences. It should also be emphasized that the success of the attention mechanism depends on the simultaneous learning of the W_*K,Q,V*_ matrices, and the word embeddings on which they operate. To the extent that the embedding vectors manage to capture the meaning of words in a large number of possible contexts, it is also possible to derive realistic interrelationships between words in a text, through simple linear transformations of these vectors. Here is the mathematical expression of the operations carried out by the attention.


(2) $$\begin{array}{l} {\vec{a}}_{i}={\text{W}}_{O}{\text{W}}_{V}\left[\begin{array}{c} {\vec{x}}_{i}\\ {\vec{x}}_{i+1}\\ \cdots \\ {\vec{x}}_{i+T} \end{array}\right]\left(\frac{1}{\sqrt{D}}{\left[\begin{array}{c} {\vec{x}}_{i}\\ {\vec{x}}_{i+1}\\ \cdots \\ {\vec{x}}_{i+T} \end{array}\right]}^{⊤}{\text{W}}_{K}^{⊤}{\text{W}}_{Q}{\vec{x}}_{i}\right) \end{array}$$

where *T* is the span of tokens preceeding the current token ${\vec{x}}_{i}$.

It should be added that this description leaves out a small additional complication: the entire token expressed as an embedded vector is actually divided into *H* portions, called heads, and the identical mechanism just described is applied separately to each head, and only in the end are the various portions re-joined. The idea is that an embedded vector combines different properties of a word, and that certain categories–for example, the tense of verbs or the gender and number of nouns and adjectives–always occupy the same portions of the vector, and therefore it is convenient to process separately the network of relationships between the separate characteristics of the various words in the text. This concise description of the Transformer clearly shows its relative simplicity compared to other DL models, and its extreme simplicity compared to the set of traditional NLP techniques required for the simulation of a wide range of natural language abilities. Finally, we must not forget that the Transformer, despite its innovative components just described, incorporates the leap forward made in transitioning from ANN to DL. The linchpin of this epochal shift is the training method. Backpropagation was the mathematical finding that gave life to the ANN of the '80s (Rumelhart et al., 1986), but it proved ineffective as soon as the networks grew in size. Already, the simple transition from a single hidden layer of a ANN to two was challenging for backpropagation (de Villers and Barnard, 1992). Limitations collapsed with DL and modern learning techniques based on stochastic gradient descent (Bottou and LeCun, 2004; Kingma and Ba, 2014). Once the limit of a single hidden layer was broken, the models began a race to have more and more layers, thus becoming “deep”. By using sophisticated mathematical topology analyses, principle reasons were identified for why “deep” networks are more efficient than “shallow” ones (Bianchini and Scarselli, 2014b,a), and of course, the Transformer greatly benefits from this. It soon emerged that a model's scale, in terms of the number of its parameters, was a crucial factor for its performance, pushing to non-theoretical, but economic limits for the cost of training. We will see the importance of the scale factor in Sections 4, 6. As with all ANN, the number of model parameters goes hand in hand with the number of samples for its training, and the corpora in use for training NLMs have grown to a significant portion of all the texts available in humanity. We have moved from the 40GB of text employed for GPT-2 to 470GB for GPT-3 (Zha et al., 2025) and 15T of tokens for Llama 3 (Grattafiori et al., 2024). Even though it is not directly related to the research on how the Transformer manages to function, it is good to highlight how the quality of the data has a far from negligible effect on overall performance. The impressive size of linguistic datasets necessitated an automatic process for controlling and filtering texts. At the same time, it was found that the subsequent refinement of NLM through examples of human preferences had a significant effect on performance (Ouyang et al., 2022). The prevalence of data care over algorithmic coding care is a common phenomenon throughout DL and is considered a sort of paradigm shift in the development of AI systems (Angius and Plebe, 2023; Zha et al., 2025).

## 3 How can neural models think?

The NLMs described in Section 2 have made a huge leap forward in language processing over the last five years (Plebe and Perconti, 2022; Min et al., 2023; Perconti and Plebe, 2025). In the meantime, not much theoretical progress has been made from the point of view of arguments challenging artificial semantics. Indeed, it has been noted that most of the current positions against the possibility of a computer acquiring meaning date back to the early arguments from the 1980s and 1990s (Perconti and Plebe, 2023). In the meantime, every minute, millions of people around the world converse with chatbots controlled by NLMs about all sorts of topics. Turing's imitation game no longer seems to be a real obstacle. There are now books on detailed philosophical conversations with a NLM (Leib, 2023). Srivastava et al. (2022) called their giant benchmark setup for evaluating the capabilities of NLMs *Beyond the Imitation Game* (BIG-bench) and assume that this type of review will be far surpassed. A team at AI21 Labs has developed a type of social imitation game in which most people are unable to distinguish whether their conversation partner is a human or an NLM (Jannai et al., 2023). In the field of research on NLMs, the main goal is to explore the intricate mechanisms that determine their functioning and their potential for cognitive inference. In the last five years or so, NLMs have made remarkable progress in the field of language processing. Nevertheless, that there is a significant lack of theoretical progress in the ongoing debate on artificial semantics. This paper does not take a definitive position on the fundamental question of whether machines can really “think”, but it argues that this is no longer the central question in the philosophy of AI. Rather, the urgent need is to understand *how* NLMs have managed to achieve a level of performance that approaches human cognition. This perspective follows an epistemological approach that emphasizes causal explanations that serve to connect the apparent simplicity of NLM algorithms with their amazing cognitive capabilities.

The passing of Turing's Imitation Game can now be considered empirically established. The performance achieved by NLMs today is so close to that of thinking human agents that it is urgent to ask how this is possible. It may be worth distinguishing here between two connotations of the term “possibility”—*a priori* (metaphysical) and *a posteriori* (empirical). The *a priori* possibility is based on the idea that there are essential properties in things and that nothing can contradict their essential properties. If one tries to imagine something that contradicts its own essential properties, she realizes that one is actually thinking of something else. There are no possible worlds in which something can have other essential properties than the one it has. However, the concept of “essential properties” is controversial. Do they really exist? If so, do they only include primary properties (such as physical properties)? Or also secondary properties, such as “hot” or “red”? Counterfactual imagination is an essential capacity for exploring metaphysical possibilities. What if water lost its chemical composition and yet retained its phenomenal properties? What if some people were behaving in such a way that they were not suspicious of their inner life and were instead a mere automaton, lacking the ability to experience the world? Ultimately, the question is whether the possibility of something can be inferred from its conceivability. Chalmers (1996) based his famous argument of philosophical zombies on this idea. Unlike movie zombies, philosophical zombies do not have a different appearance than usual. In fact, they are indistinguishable from their sentient counterparts. But unlike their sentient counterparts, they feel nothing. If such creatures are conceivable, perhaps they are also possible. But, if they are possible, then physicalism is false. Physicalism is the view that every aspect of the mind, including consciousness, can be entirely explained by physical processes; the conceivable existence of zombies—beings physically identical to us but lacking any subjective experience—contradicts this claim. One might object that possibility can be inferred from mere conceivability, and that the mere possibility of something does not have the power to falsify a theory about the real world. But that is not the point we are trying to make.

This paper rather considers *a posteriori* possibility, which depends on whether something has actually happened. In a sense, it is implicitly based on the famous dictum of medieval metaphysics: *Ab esse ad posse valet*. Taking the a posteriori possibility into account brackets the “can comes first objection”, on the grounds that sufficient empirical evidence exists to justify questioning how this phenomenon might have occurred or, at the very least, to request that the interlocutor concede this point “for the sake of argument.” The question of empirical possibility sounds something like this: If certain things actually happened, how was it possible for them to have happened? Empirical possibility, which is based on *a posteriori* observations, is about unraveling the *mechanism* by which certain events came about. For example, if we consider the human achievement of powered flight, we might ask about the empirical possibility of such an achievement. The answer involves strategies such as mimicking the flight of animals, a deeper understanding of the laws of physics, and the consideration of the physical parameters that determine the “flight”, whether natural or artificial. In this perspective, “flight” is no longer just something that birds can do, but a *function* can be attributed to any object that conforms to the “laws of flight”. In this broader sense, a bird, a bat, a jet plane and a hang glider are all objects capable of flight, as they follow the same rules and their behavior is based on the same laws. Flight is therefore no longer understood as an exclusive function of birds or certain mammals, but as a “mechanism” used by certain animals in nature, selected in the course of evolution, and which can even be relied upon to achieve an artificial version of flight. It is similar with the mind. Intuitively, the mind is exactly what characterizes humans, like flight for birds. Or at least a component of it, such as self-consciousness, language, or creativity.

Cognitive science has contradicted this common sense intuition. By investigating how a particular cognitive function, such as vision, works, an increasing understanding of the underlying cognitive mechanisms has been gradually uncovered. The cognitive mechanisms of vision were then transformed into a computational architecture, which eventually made artificial vision possible. By extending this approach to the various cognitive abilities, the dream of an “artificial intelligence” somehow became a reality. Artificial systems were then used to discover new mechanisms about natural cognitive systems: given a function, implemented in an artificial system by a specified mechanism, an hypothesis is advanced that the same, or similar, mechanism realizes the same function in the natural system. Just as “flying ”is something that occurs in both natural and artificial forms, “thinking” is something that occurs in different ways, including artificial thinking. By disciplining ethnocentrism and anthropocentrism, we may also be more willing to be liberal toward artificial intelligence. If the mind is a consistent set of computational architectures, then the way humans have their minds and the way machines are endowed with theirs are actually two variants of the same phenomenon. Cognitive science has long hypothesized that thought is a form of computation that occurs through meaningful representations of the world. The idea that mental representations serve as tools for people to navigate the social and physical world, and thus guide their behavior, has persisted in the philosophy of mind for centuries. These representations are endowed with intentionality, which means that they refer to specific contents that relate to states of affairs. Consequently, these representations convey meaning by signaling the information they contain about various aspects of the world. This way of reasoning holds if we restrict it to the human mind or analogous animal species. However, when we consider artificial intelligence, a profound change emerges.

In the course of its historical development, computationalism has encountered challenges posed by alternative cognitive science paradigms such as embodiment and enactivism. At the same time, the computational theory of mind has become increasingly close to the processes of the human brain, claiming that a computer equipped with an appropriate program can think and process meaningful representations. Turing (1950) originally introduced this concept with the idea of engaging computers in ordinary conversations to assess their cognitive abilities – a challenge that was later taken up by researchers in the field of artificial intelligence. The concept of thinking machines has led to considerable debate and skepticism. Dreyfus (1972, 1992) and Searle (1980) in particular found the notion of thinking machines unsettling. Even among the proponents of the computational theory of mind, there were those who harbored doubts about attributing meaning to computers (Dretske, 1985). The quest for a semantics for computers and the related field of artificial intelligence gave rise to lively debates in the 1980s and 1990s (Haugeland, 1985; Dennett, 1985; Pylyshyn, 1987; Harnad, 1989, 1990; Dennett, 1998; French, 2000). The philosophical discussion on the topic subsequently experienced a general decline in interest, albeit with some exceptions (Preston and Bishop, 2002; Chalmers, 2010; Plebe and Perconti, 2013). At the beginning of the 21st century, however, disillusionment spread in the ranks of AI proponents, as AI was unable to keep up with human cognitive abilities in critical tasks. This led to fatigue and division within the AI community and raised questions about the future of the field. The question of whether machines can really “think” remains unresolved, but it is evident that NLMs have come remarkably close to mimicking human cognitive processes. The present investigation takes an epistemological standpoint and attempts to uncover the *causal explanations* for the exceptional cognitive abilities of NLMs. This is done by analysing the current literature on NLMs and their functioning under the light of the problem of scientific explanations in cognitive science (Haugeland, 1991; Bechtel, 2008).

## 4 Functional analyses

One first kind of explanations of NLM behaviors can be considered *functional* in that it tries to adapt the same methodologies used for human subjects in tracing the emergence of a series of cognitive and linguistic phenomena. There is indeed a growing body of research targeting specific cognitive and psycholinguistic functions in NLMs, functions that support the ability to think shown by the models. This trend is related with the increasing prominence of NLMs in the field of experimental psychology. The ease and flexibility of using NLMs align well with the textual nature of many tests in this field. The alliance between NLM and psychology has a dual perspective. NLMs are potential tools that assist psychology in studying the human mind, as discussed (Demszky et al., 2023). Alternatively, it is psychology, with its own tools, that aids in the analysis of NLMs, a tendency dubbed *machine psychology* (Hagendorff, 2023). It ecompasses examples where NLMs are equated to human subjects in tasks and tests, being used as *social simulacra* (Park et al., 2022) or *silicon samples* (Argyle et al., 2023). Let us see three typical psychological functions that has been searched using NLMs as if they were humans, with one caveat: it is a quite debated topic whether psychological tests developed for human subjects can be equally applied to NLMs to assess whether the latter actually possess the same psychological functions of the former. For instance, Löhn et al. (2024) stress how assesment procedures for psychological tests usually go through standard validation processes over the years; similar standardization processes are lacking when evaluating NLMs tests.^2^ However, this paper does not want to take part to such a debate on the validity of current machine psychology, which certainly needs improvements, but rather to highlight how one tentative explanation of NLMs linguistic abilities can be put on a par with functional explanations in cognitive science. Let us see.

*Theory of Mind*: in literature, the capacity to adopt other person's mental perspective and to anticipate their behavior is discussed through various expressions, including Theory of Mind (ToM), mentalization, mindreading, and social cognition (Apperly, 2010; Heyes and Frith, 2010). Although the term “mindreading” may seem somewhat unusual, it is widely used as a neutral expression encompassing the set of processes that allow us to represent others' mental states (Barlassina and Gordon, 2017). This capacity forms the basis not only of linguistic abilities and communication in general, but also of self-awareness, empathy, and moral judgment (Zhang et al., 2012). The widespread use of the term “theory of mind” is due to Premack and Woodruff (1978) in cognitive ethology (Rosati et al., 2010), and later to Baron-Cohen et al. (1985). In past decades, theory of mind was generally conceived as a type of folk knowledge–specifically, knowledge pertaining to the mind (folk psychology, like folk physics, folk biology, etc.). The idea that the mentalistic interpretation of behavior is based on the ability to model the mental states of others has been challenged by simulation theory (Goldman, 2006), which offers an explanation grounded in the capacity to internally and directly simulate the inner life of other individuals. What is controversial is how people arrive at representing others' mental states. On one side, proponents of the Theory-Theory of mindreading (TT) hold that a tacit psychological theory underlies the ability to represent and reason about others' mental states, arguing that mentalization is a theory-driven, information-rich process. On the other side, proponents of Simulation Theory (ST) argue that representing others' mental states is an information-poor process based on the ability to put oneself in others' shoes and ask what we would do in their place. TT holds that when we represent other people's mental states, we consult a tacit but systematic set of propositions about how the mind works. According to ST, however, no theory, not even an implicit one, is needed, since the ability to represent others' mental states is simply a matter of directly adopting the other's perspective through simulation and by projecting ourselves into their situation. In other words, to interpret others' behavior we engage in a process of mental simulation by imagining ourselves in their situation and using our own cognitive mechanisms to anticipate or explain their thoughts, emotions, and decisions (Iacoboni, 2014). As a result of the debate between Simulation Theory and Theory-Theory, a hybrid version has emerged (Röska-Hardy, 2008; Gallagher, 2015; Venter, 2024). The “hybrid account” suggests that understanding others' minds involves both simulation and the application of a form of theory of mind that incorporates folk psychology. In this sense, the term “theory of mind” has shed its initial commitment to theory-theory and is equally parsimonious, from an epistemological standpoint, as broader concepts such as mindreading or social cognition. It should also be considered that the “theory of mind,” as specified, consists of two main components, namely low-level mindreading (emotional contagion, gaze following, etc.) and high-level mindreading (counterfactual reasoning, stream of consciousness, etc.) (Goldman, 2006). While low-level mindreading is predominantly non-linguistic, high-level mindreading is largely mediated by language. For example, silent verbal reasoning is often used as an internal logical space for behavioral prediction. These considerations lead us to appreciate that, although linguistic competence and theory of mind are not entirely overlapping–and indeed in some cases operate independently–in general, investigating the linguistic mediators of social cognition is a highly promising perspective. This is the primary reason why ToM is included in our short list of functional analysis of NLM.

Kosinski (2023) at Stanford developed a strategy for administering the classical false-belief tasks, widely used to test ToM in humans, to several versions of GPT. Let us recall that the false-belief task amounts to introducing a story to test the ability of the listener of understanding that the protagonist may have a belief the listener knows to be false. The claim is that whereas ToM is certainly not a function specified and implemented in NLMs, it nonetheless emerges from the network while being trained to process human language. This is in line with research in evolutionary psychology and linguistics maintaining that ToM emerged at a certain stage of language evolution as a biological adaptation (Milligan et al., 2007). Indeed, ToM is an essential feature to interpret sentences containing mental predicates (such as think, believe, desire etc.) and, consequently, to generate new related sentences. The experiments of Kosinski revealed a level of ToM in line with 3-year-old children for GPT-3.5, and in line with 7-year-old children for GPT-4. A particularly notable aspect of Kosinski's experiments is the relationship highlighted between the scales of the Transformer and evidence of ToM. Models with less than 100B parameters do not possess any ToM, GPT-3.5 with 175B parameters has a level of ToM comparable to a 3-year-old child, and GPT-4 with over 1 trillion parameters is comparable to a 7-year-old child. We will see that scale also plays a significant role in the relationship between Transformer and the brain (Section 6). A conclusion that is far from definitive, the topic of ToM in NLM soon became the subject of lively discussion (Brunet-Gouet et al., 2023; Holterman and van Deemter, 2023; Leer et al., 2023; Ma et al., 2023; Marchetti et al., 2023; Trott et al., 2023). The focus here is not on whether or not NLMs possess ToM. This case is exemplary, for the economy of this article, as a functional methodology for accounting for the linguistic abilities of NLMs.

Other mental functions which can contribute to explain NLMs behaviors are quickly reported in the following.

*Discourse entity tracking* is another fundamental capacity in humans for linguistic social communication. It encompasses several abilities, including the recognition of new discourse entities when introduced, coreference resolution, namely associating different linguistic expressions with the same entity, and tracking the state changes of the introduced entities. Kim and Schuster (2023) setup a series of experimental tasks, where entities undergo several changes during a discourse, to test GPT 3, GPT 3,5, and FLAN T-5 against discourse entity tracking abilities. Models are trained over datasets containing linguistic descriptions of boxes and objects which can be loaded, taken, or moved from one box to another one. The task consisted, given an initial description of the state of affairs, and a set of operations on the objects in the boxes, to correctly describe what objects a given box contains. Models were prompted by defining the task, introducing two task examples, describing the initial state of affairs, and providing a sentence of the form *Box N contains …* to be completed to solve the task. The authors have developed a completely new dataset for these experiments, in order to prevent state transitions of entities from following a pattern already present in the pretraining data of the models. This is a necessary methodological precaution, which, however, precludes the possibility of a direct comparison with the tracking accuracy of human subjects. After all, this is not the primary interest of the work, but rather to verify whether this kind of competence – notoriously fundamental in human language – could be found to some extent in the models. In the first of the experiment described (Kim and Schuster, 2023), only GPT-3.5 showed considerable discourse entity tracking abilities, outperforming both GPT-3 and FLAN T-5. Prediction accuracy of GPT-3.5 decreased as the number of operations on the objects in the boxes increases, from more than 90% of answer accuracy after one operation to more than 25% after seven operations.

*Property induction* is another, core, function displayed in human inductive reasoning which has been found to be realized also in NLMs. Property induction is the ability to extend a property that is shared by some categories, to different categories, when appropriate. Han et al. (2024) performed two kinds of experiments in order to compare the results of property induction tasks by humans and by GPT-3.5 and GPT-4 models, in three category domains, namely mammals, birds, and vehicles. The first experiment is about selecting which, between two inductive arguments, is stronger; the second experiment involves rating the strength of a single arguments. The authors have constructed the tasks by reproducing a series of known psychological phenomena that either strengthen or weaken property induction. One such phenomenon is similarity. For instance, starting from the premise that cats have sesamoid bones, when asked if lions also have sesamoid bones, the responses are more positive compared to asking if giraffes have this type of bone. The first argument is stronger than the second because cats are more similar to lions than giraffes. An example of a phenomenon that weakens property induction is non-monotonicity, which occurs when an additional premise involves a different entity from the category in question. Non-monotonic reasoning is a major form of reasoning used by humans that contrasts with standard deductive reasoning (Brewka et al., 1997). For example, the premises that both cats and lions have sesamoid bones would strongly suggest that all mammals have them. But adding the information that crocodiles also have sesamoid bones makes the argument weaker. Results shows that while GPT-3.5 performs quite poorly, GPT-4 correctly evaluates stronger arguments in a similar way humans do, except for non-monotonicity, respect with which bot GPT-3.5 and GPT-4 fail in identifying the stronger argument.

Again, what counts for the present analysis in not whether NLMs outperform or not human linguistic behaviors, but rather that NLMs are studied in terms of functions and the extent to which they implement them. Explaining the behaviors of a system in terms of the functions the system implements was the aim of Nagel (1961)'s initial account of *functional explanation*. His definition was behavioral: for a biological or social system to possess a given function means behaving in a certain way. Similarly, for a NLM to possess psycholinguistic functions means, just to consider the three provided study cases, being able to solve the false belief task, to track discourse entities, or to induce object properties. If Nagel's aim was to reduce the functional explanations one employs in life or social sciences to the nomological-deductive ones used in physics, properly levering on the behavioral definition of function, Cummins (1975, 1983) is more interested in explaining the capacities of complex systems, such as cognitive systems. This is done through what is known as the *functional analysis*: the general capacity of a system is explained in terms of the components of such a system and of the functions realized by those components. It is essential to show how the functions of the components, together with their causal organization, bring about the general system capacity. For instance, the capacity of the circulatory system can be explained in terms of some system components, such as arteries, veins, and the heart. Each component has a function contributing to the general function: the function of the heart is to pump blood through the arteries.

Since each component has got a function, it can be examined in terms of its sub-components and sub-functions as well. In the case of the heart, those may be atria, ventricles, valves, myocardium, and so forth, each with its proper subfunction. The functional analysis can still proceed by analysing sub-components and their sub-functions in terms of their components and related functions. The functional analysis halts either when one reaches a successful explanatory level or when one reaches the structural, physical, level of components that can be explained mechanistically, without reference to functions and sub-components. The kind of explanation of linguistic abilities of NLMs current research is trying to attain, certainly involves a general function being analyzed into sub-functions. Linguistic capacities are being explained in terms of sub-capacities such as possessing a ToM, or being able of discourse entity tracking and property induction. And each of this is analyzed in terms of functions allowing a model to possess the given sub-capacity. For instance, having a ToM is analyzed in terms of the ability to solve some different false belief tasks; and solving a false belief task implies correctly answering to 16 prompts covering 8 different scenarios. Clearly, a function analysis may fail: a capacity may be wrongly decomposed into sub-components and sub-functions; for instance, those arguing against the ascription of a ToM to NLMs would agree that their linguistic capacities are being wrongly analyzed in sub-functional terms (the ToM). Again, we are not arguing in favor or against given psychological functions for NLMs, but rather highlighting how these initial attempts in machine psychology amount to a (successful or failing) functional analysis.

However, Cummins is very clear in stating that performing a functional analysis in terms of sub-functions and their sub-sub-functions is not enough for the functional analysis to be considered an explanation (Cummins, 1983). Each sub-function must be associated to a system component, the *causal role filler*; in other words, for each identified sub-function one is required to identify the physical structure or *mechanism* implementing that function. This can hardly be done with NLMs which, as any other DL architecture, are highly opaque and uninterpretable (Lipton, 2016). What is not known are the data pattern a network isolates to provide the resulting output and, especially, the components, i.e. neuron networks, being activated to output the given result. The search for internal mechanisms within Transformers that underpin their linguistic successes undoubtedly benefits from techniques that have been refined within the field of study known as XAI (*Explainable Artificial Intelligence*) (Zednik, 2021; Langer et al., 2021; Hassija et al., 2024). However, it's important to highlight the fundamental differences between XAI and the endeavor advocated here. The XAI perspective is primarily pragmatic, and the exemplary case studies of interest concern exceptions, whenever an AI system makes mistakes, and there is interest in identifying the causes of the failure. Our perspective is instead theoretical. Understood, even the cases where an AI system fails can be revealing about its overall functioning. However, the perspective taken here primarily aims to make sense of how models manage to work in ordinary cases, and to work so well. Let us see.

## 5 Thinking about mechanisms

The studies that, from our survey, appear to be engaged in researching mechanisms capable of explaining aspects of the Transformer, use a variety of tools, which can be classified as follows:

- *Circuit discovery*, tools able to extract from NLMs circuits with distinct functionality, an example is the ACDC (*Automatic Circuit DisCovery*) (Conmy et al., 2023);
- *Localization*, strategies for localizing neurons or group of neurons in NLMs that are responsible for specific basic tasks, like classifying the tense of a verb; an example is *sparse probing* (Gurnee et al., 2023);
- *Visualization*, like *AttentionViz* (Yeh et al., 2023), that generates colored 2D graphs of principal components of the attention matrices for key and query of the same token; or the graph representation, with colored edges, of the attention values for a prompt (Katz and Belinkov, 2023);
- *Conversion*, between a human-readable programming language for composing simple primitive language tasks, and an equivalent Transformer model; examples are RASP (*Restricted Access Sequence Processing Language*) (Weiss et al., 2021; Lindner et al., 2023); and other way round, like *Transformer Programs*, simplified Transformer models that can be converted into a discrete, human-readable program (Friedman et al., 2023).

Not all studies analyse NLMs as a whole, some focus on one part of the model only: *attention* (Tenney et al., 2019; Clark et al., 2019; Snell et al., 2021; Mittal et al., 2021; Katz and Belinkov, 2023; Yeh et al., 2023); *feed-forward layers* (Geva et al., 2021, 2022; Merullo et al., 2023; Huben et al., 2023); *embedding* (Hewitt and Manning, 2019; Mickus et al., 2022).

Another dimension along which the studies differentiate is the phase at which the NLM is investigated. Prediction is the phase on which most studies focus their attention; while some analyse the training phase (Snell et al., 2021; Tian et al., 2023); and others target *in-context learning* (ICL). Thanks to ICL, NLMs can perform cognitive tasks that they previously could not, or only to a limited extent, after seeing a few examples in the prompt. This is a phenomenon that has recently converged a significant number of works (Garg et al., 2022; Olsson et al., 2022; Abernethy et al., 2023; Von Oswald et al., 2023; Wibisono and Wang, 2023; Zhang et al., 2023), partly due to the fact that it is a well-defined phenomenon that develops over a few interactions, unlike the billions of steps during full training. Although these works often claim to have the discovery of the mechanisms behind ICL as their objective, the results are often configured as mathematical descriptions rather than mechanisms in the proper sense. For example, Abernethy et al. (2023) demonstrate that, under suitable conditions, ICL in a Transformer corresponds mathematically to the hypothesis of a tokenized sparse linear regression. Similarly, Guo et al. (2023) demonstrates the equivalence between ICL in the Transformer, under certain simplified conditions, with an optimal ridge predictor; and the equivalence with Bayesian Model Averaging (Zhang et al., 2023) .

As a whole, all the surveyed studies provide a scattered and modest picture of potential mechanisms explaining how NLM can work. Yet they are important as pioneering investigations in this direction. Here we intend to focus only on one study, due to Elhage et al. (2021), one of the first to venture in the direction of mechanism research, and even today, one of the most profound and revealing. His strategy is not dissimilar from that of other studies, starting from the fewest possible components of a Transformer, in its minimal implementation, looking for possible elementary mechanisms, and the types of phenomena they are capable of producing. The two models analyzed are a single layer with attention only, and a stack of two layers, still with attention only without feedforward layers. Two basic independent components have been identified: the OV (*output value*) circuit made by the matrix **W**_*O*_**W**_*V*_, and the QK (*query-key*) circuit made by the matrix ${\text{W}}_{\text{Q}}^{\text{T}}{\text{W}}_{K}$, whose matrices are computed as follows, in according with Equation 2:


(3) $$\begin{array}{l} {\text{W}}_{OV}={\text{W}}_{O}{\text{W}}_{V} \end{array}$$

(4) $$\begin{array}{l} {\text{W}}_{QK}={\text{W}}_{Q}^{⊤}{\text{W}}_{K} \end{array}$$

In the one layer model the most significant phenomenon is the *copying* algorithm, that performs this simple mapping:


(5) $$\begin{array}{l} \left[\ldots ,a,b,\ldots \right]\to a \end{array}$$

in practice previous tokens are likely to be the next predicted. It is the OV ciruits that predisposes things so that tokens, if attended to by the head, become probably the next token. The QK circuit attends back to all tokens which could plausibly be the next token. Thus token, whenever their place is plausible, are copied. When moving to a two-layer model, additional components come in place, called K-, Q-, V-Compositions. These components account for how much information query, key or value vectors of a second layer attention read from a given first layer attention. They are computed as follows:


(6) $$\begin{array}{l} \xi_{K}=\frac{\|{\text{W}}_{QK}^{\left(2\right)}{\text{W}}_{QK}^{\left(1\right)}|{|}_{F}}{\|{\text{W}}_{QK}^{\left(2\right)}|{|}_{F}\|{\text{W}}_{QK}^{\left(1\right)}|{|}_{F}} \end{array}$$

(7) $$\begin{array}{l} \xi_{Q}=\frac{\|{{\text{W}}_{QK}^{\left(2\right)}}^{⊤}{\text{W}}_{QK}^{\left(1\right)}|{|}_{F}}{\|{{\text{W}}_{QK}^{\left(2\right)}}^{⊤}|{|}_{F}\|{\text{W}}_{QK}^{\left(1\right)}|{|}_{F}} \end{array}$$

(8) $$\begin{array}{l} \xi_{V}=\frac{\|{{\text{W}}_{OV}^{\left(2\right)}}^{⊤}{\text{W}}_{OV}^{\left(1\right)}|{|}_{F}}{\|{{\text{W}}_{OV}^{\left(2\right)}}^{⊤}|{|}_{F}\|{\text{W}}_{OV}^{\left(1\right)}|{|}_{F}} \end{array}$$

where ||·||_*F*_ is the Frobenius norm, and the superscripts ^(1)^ and ^(1)^ specify to which layer the matrix belongs.

The composition quantities ξ_*K,Q,V*_ allow the discovery of a a novel phenomena that was not present in one-layer, and emerge in the two-layer model:


(9) $$\begin{array}{l} \left[\ldots ,a,b,\ldots a\right]\to b \end{array}$$

where the present token is searched over the current context, and if it is found, then the token that was next in the context is predicted. This phenomenon is called *induction head*. It relies in part on the copying phenomenon seen in one-layer models, but it also requires an additional one called *prefix matching*, the capacity to compare the current token with every preceding token and look for places where they are similar. This is possible thanks to the K-composition in the QK circuit of the second layer.

Let us now ask whether induction head can be considered a discovered mechanism of some sort, and whether its description amounts to the *mechanist explanation* of some given phenomenon. The issue of mechanists explanation, in its contemporary account, traces back to Machamer et al. (2000) original paper wherein a mechanism is defined as a set of “entities and activities organized such that they are productive of regular changes from start or set-up to finish or termination condition” (p. 3). In the context of the explanation of biological and neurocognitive phenomena, mechanisms are defined by a set of entities that, by entertaining causal relations, bring about a given phenomenon, i.e. the *explanandum*. For instance, neurotransmission can be explained in terms of a set of entities, say axons, dendrites, synapses, neurotransmitters, vesicles, receptors, and their causal relations, such as the fusion of a vesicle to the axon membrane, the release of the neurotransmitter in the synaptic cleft, and reception by the dendrite receptors. Advancing a mechanist explanation of a phenomenon amounts to describing a mechanism of this sort that brings about the phenomenon. Mechanisms are usually delimited by starting and finishing conditions; in the case of neurotransmission those may be respectively identified with the depolarization of the axon membrane and the deactivation of the neurotransmitter in the postsynaptic neuron.

Induction head can be certainly understood as a mechanism defined by a set of entities, namely neuron layers, embedded tokens, the K-, Q-, V- vectors, circuits, matrices, and their causal relations, i.e. the matrices operations on vectors. Those causal operations bring about the main phenomenon at the basis of the induction heads, that is, guessing a next token in a context, when it satisfies the conditions of Equation 9. While the composition quantities ξ_*K,Q,V*_ computed in Equations 6, 7, 8 allows the detection of this phenomena, the group at Meta AI lead by Léon Bottou went further and described in mechanistic way the conditions that induce induction heads (Bietti et al., 2023). This was possible at the price of drastic simplifications, in addition to those already imposed by Elhage and co-workers. The query matrices were fixed ${\text{W}}_{Q}^{\left(1\right)}={\text{W}}_{Q}^{\left(2\right)}=\text{I}$ so that the key matrices ${\text{W}}_{K}^{\left(1\right)}$ and ${\text{W}}_{K}^{\left(2\right)}$ play the role of both key and query matrices, and the matrices ${\text{W}}_{V}^{\left(1\right)}$, ${\text{W}}_{O}^{\left(1\right)}$, and ${\text{W}}_{V}^{\left(2\right)}$ did not change during training. It should be noted here that the induction head actually corresponds to a kind of *multi-level mechanism*, in that it results from the interaction of the copying algorithm and prefix matching, which can be seen as phenomena resulting from lower level mechanisms. This is in line with the multi-level analysis of neurocognitive mechanisms (Craver, 2016), according to which given a mechanism *M* conventionally ascribed to level 0, an entity *X* of *M* can be analyzed at a lower mechanist level −1 which entities and activities constitutes *X* at level 0, and at an upper mechanist level +1 in terms of the contribution given by *X* to the upper level mechanism. Whereas it is clear that induction head as a mechanism can be analyzed as the composition of lower levels mechanisms, namely the copying algorithm and the prefix matching, one may wonder whether there are upper level phenomena to which induction head contributes. It has been speculated that induction head is the key mechanism for the higher level phenomenon ICL, seen before. ICL is clearly at the basis of the broad capacity of “thinking” one may ascribe to NLMs. Olsson et al. (2022) provided compelling arguments for linking induction head to in-context learning. The main argument is the co-occurrence of ICL and induction head during training of NLMs, an event relatively easy to detect, because of a sudden and dramatic collapse of the loss for ICL during training. This event coincides with the emergence of induction head in the second layer of the model. The second argument put forth by the authors is interesting primarily for reinforcing the result, but also for its explanatory strategy. Indeed, it fully embodies a significant variant of mechanism known in the philosophy of science as *interventionism* (Woodward, 2003). This methodological approach preserves the mechanistic principle of identifying parts within a system and their role in producing a phenomenon, but it aims to discover causal relationships through intervention on certain parts, and observing modifications of aspects of the phenomenon which are exclusively due to the intervention made. Olsson and her colleagues injected a small modification into the Transformer, dubbed the *smeared key*, that artificially facilitates the emergence of the induction head. The model with this modification demonstrated a dramatic change in the emergence of ICL, precisely coinciding with the anticipated expression of the induction head.

One may ask whether ICL is actually the higher level mechanisms to which induction head, as a mechanism, contribute. ICL, as a phenomenon, refers to a system capacity, viz. the capacity of predicting tokens later in a context. It has also been stated that such capacity is deemed to be at the basis of the general capacity of NLMs of performing human-like linguistic behaviors. In other words, in-context learning seems to be one sub-function of a NLM general function and to fall under the functional analysis approach recalled examined in the previous section. Nonetheless, ICL also refers to tokens, context, mappings from tokens to tokens etc which are all component of the mechanisms explaining the good performances of the network. Mechanisms not specifying all causal role fillers, but also containing unspecified functional roles (Machamer et al., 2000) *mechanism sketches*. More specifically, Machamer distinguishes a mechanism, as previously defined, from a *mechanism schema* which is “a truncated abstract description of a mechanism that can be filled with descriptions of known component parts and activities” (p. 15), and from a mechanism sketch, “an abstraction for which bottom out entities and activities cannot (yet) be supplied or which contains gaps in its stages” (p. 18). Piccinini and Craver (2011) have it that mechanisms in neuroscience are organized in a level hierarchy, where lower levels provide full-fledged, bottoming out, mechanisms wherein all entities and activities have been specified. As one goes up in the hierarchy, some of the entities and activities are left unspecified, forming sketches of mechanisms. The authors argue that providing mechanism sketches of this sort is akin to a functional explanation wherein bottoming-out entities and their causal relations are replaced by functional roles. This, in turns, allows to understand how functional explanations one finds in psychological theories can be reduced to the mechanist explanations used in neuroscience. Indeed, functional explanations are, according to this view, mechanism sketches which can be reduced to full-fledged mechanisms by specifying the bottoming-out causal role fillers for the functional roles.

We finally want to mention a strategy that lies halfway between the mechanistic and the functional explanation, and draws distant inspiration from the concept of sparse coding (Olshausen and Field, 2004), that is, the ability to encode, with a certain number of neurons, a much higher number of features. In its proposition by Huben et al. (2023), it is implemented through sparse autoencoders.


(10) $$\begin{array}{l} \vec{z}=f\left(\text{M}\vec{a}+\vec{b}\right) \end{array}$$

(11) $$\begin{array}{l} {\vec{a}}^{\prime }={\text{M}}^{⊤}\vec{z} \end{array}$$

where *f*(·) is a non linear squeezing function, typically ReLU, ${\vec{a}}^{\prime }$ is the approximate reconstruction of $\vec{a}$, and $\vec{b}\in {\mathbb{R}}^{RD}$, **M** ∈ ℝ^*RD*×*D*^, are learned, with *R* the sparsity factor.

The inputs of the autoencoder are vectors $\vec{a}$, like those in Equation 2, while the latent vector $\vec{z}$ has a greater dimension, fixed by the parameter *R*. By training the autoencoder on a wide set of NLM activations, a feature dictionary is formed in matrix M, each represented sparsely by the population of neurons used for training. Sparse autoencoders are considered here to be halfway between functional and mechanistic analysis as they aim to identify a set of features in a holistic way, without resorting to detailed interpretation of the model's circuit mechanisms. However, the features do not correspond to functions in a cognitive sense, as they are nothing more than numerical vectors in themselves. In fact, a crucial part of the method consists of reaching an intelligible interpretation of these features (Bills et al., 2023), a task that remains critical (Huang et al., 2023). The bridge with the mechanistic interpretation is further corroborated by the ability offered by the sparse autoencoders to exploit the feature dictionary by tracking the possible causal effect of the activation of a specific feature on others, and identifying the circuits along which causal links of this kind occur (Bricken et al., 2023; Templeton et al., 2024). The linguistic abilities of NLMs can be explained on the one hand through a functional analysis that identifies their capacities, as described in the previous section, by isolating sub-functions and their relationships. On the other hand, they can be understood by examining the inner mechanisms of NLMs, as exemplified in this section, recognizing different layers of mechanisms and linking them back to higher level sub-functions of the network, such as in-context learning and few-shot learning.

It follows that the explanatory strategies one adopts to understand the human-like linguistic abilities of NLMs can be put on a par with the explanatory strategies used to explain the human cognitive system, namely by advancing functional, psychological, explanations on one side, and mechanist, neuroscientific, explanations on the other side, and by trying to reduce the former to the latter through a hierarchy of bottoming-out mechanisms. The idea of looking at natural cognitive systems to understand the artificial ones revers the traditional, methodological, setting of simulative AI, wherein the artificial system is used to understand the mind. This point is developed in the next section, by examining a third kind of explanation for NLMs.

## 6 Looking at the brain

A third kind of explanations, here called *simulative*, investigates relationships between NLM structures and brain structures, through Functional magnetic resonance imaging (fMRI), when engaged in the same linguistic task. It is a surprising line of research, unexpected even for its own protagonists. Apart from the generic inspiration from biological neurons for artificial neurons, there is nothing specific in the transformer mechanisms that has been designed with the brain language processing in mind. Despite the fragile premises, this path has been able to benefit from a similar line of research on the similarities between visual processes in deep learning networks and in the cortex, now well-established (Khaligh-Razavi and Kriegeskorte, 2014; Güçlü and van Gerven, 2015; Eickenberg et al., 2017). However, while the structure of deep convolutional neural networks offered a hierarchical structure that vaguely recalls the ventral visual pathway of the cortex, none of this is found in Transformers. However, early results show surprising correlations between activation patterns measured in the NLM and in the brain, and some analogies in the hierarchical organizations in NLM and cortex (Caucheteux and King, 2022; Caucheteux et al., 2022, 2023).

Charlotte Caucheteux in collaboration with Meta AI, did several experiments to examine correlations between NLMs prompted with stories and brain activities of subjects listening to the same stories. To measure how much Transformers and the brain show similarities, a metric consolidated from experience with the visual system, called the *brain score*, is used (Schrimpf et al., 2020a,b). Using the nomenclature of these works, we call neuroid either neural recordings or model activations. Given a certain input stimulus, for each target neuroid, we have its actual response $\vec{y}$, and a ${\vec{y}}^{\prime }$ obtained with a linear transformation of the response of the source neuroid. The brain score for each neuroid is obtained from the Pearson correlation coefficient:


(12) $$\begin{array}{l} r=\frac{\sum \left(\vec{y}-\vec{\mu }\right)\left({\vec{y}}^{\prime }-{\vec{\mu }}^{\prime }\right)}{\sqrt{\sum \left(\vec{y}-\vec{\mu }\right)\left({\vec{y}}^{\prime }-{\vec{\mu }}^{\prime }\right)}} \end{array}$$

where the summation is over a set of stimuli, and $\vec{\mu }$ is the average over all stimuli. The value *r* is then averaged for all neuroids of a brain region, or a layer of the model.

It should be emphasized in Equation 12 the perfect symmetry of the neuroids, and thus between brain data used as predictive of the model, and vice versa, which leads us to qualify the kind of epistemology at work here as *co-simulation* (Angius et al., 2024), a concept that will be detailed further on.

In a recent study (Caucheteux and King, 2022) a dataset of 204 native Dutch speakers reading 400 distinct sentences was used. In Caucheteux et al. (2022, 2023) the more recent and complete Narratives dataset (Nastase et al., 2021) is used. It is made of 345 subjects listening to short 27 narratives. The used NLM is a 12-layer open GPT-2 provided by HuggingFace. The main aim of Caucheteux is to evaluate the similarity in language processing between NLM and humans (Caucheteux et al., 2022), for this purpose among the 27 stories of the Narratives dataset only the seven stories for which subjects were asked to answer a comprehension questionnaire at the end are used. The highest brain scores are found in the middle layers of GPT-2, correlating with areas as the auditory cortex, the anterior temporal area, and the superior temporal area. Most of all, the brain scores of GPT-2 are highly correlated with the level of language processing in the subjects. In another study (Caucheteux et al., 2023) the aim is at explaining one main difference occurring between NLMs and brain language processing, namely that while NLMs are trained to guess the most probable next word, the brain is able to predict sensibly longer-range words. In this case all the 27 stories in the Narratives dataset, listened by 304 individuals, are used. In addition to the localization of areas with the best brain scores, the authors evaluated whether considering longer-range word predictions in the Transformer produces higher brain scores. The result was a positive answer for a range of up to 10 words, with a peak for a 8 word-range. Even more intriguing are the results of the relationship between levels in GPT-2 and the hierarchical scale in the cerebral cortex. The associative cortices are best modeled by the deeper layers, while the lower-level language areas, such as Heschl's gyri/sulc, are best predicted by the first layers of the NLM.

Similarly, Kumar et al. (2023) at Princeton Neuroscience Institute investigated possible correlations between the individual attention heads in the Transformer, and brain areas when listening to stories. They used a simple model, BERT, with 12 layers and 12 attention heads, and applied Principle Component Analysis to the 144 model activations along the story, correlating them with brain areas obtained through fMRI. In both studies, Transformer based NLMs are used to model and predict activation patterns in the brain observed through fMRI, in order to collect additional evidence on the brain areas involved in specific linguistic tasks.

Both systems, the ANN and the human brain, are subjected to the same task, which is to process acoustic signals (the listened story) in order to process language understanding. The artificial system is used to predict behaviors (brain activations) of the natural one. This methodology is at the basis of the *simulative method* in science (Winsberg, 2010; Durán, 2018), according to which a target, natural, system is modeled by a mathematical model, which is then implemented in a computational one, usually a simulative program, the latter is executed and executions provide predictions of the target system behaviors. In the realm of cognitive science, the simulative method amounts to implementing an artificial system, either a robot or a computer program, aimed at testing some given cognitive hypotheses on a natural cognitive system (Datteri, 2017). Given a cognitive function and a hypothesis concerning the identification of a mechanism able to implement that function, the simulative or “synthetic”method in cognitive science aims at constructing an artificial cognitive system implementing that mechanism for the given function in order to compare the behaviors of artificial and natural systems. In case the displayed function of the simulative system matches with the behaviors of the simulated system, the initial hypothesis concerning how the function under interest is realized in terms of the implemented mechanisms is corroborated. Once corroboration is achieved, simulations on the artificial system are used to predict, and explain, the future behaviors of the natural system. In order to explain past or future behaviors of natural cognitive systems, one may advance both functional and mechanist explanations based on capacities and mechanisms in the artificial system reproducing that behavior. The synthetic, simulative, method in cognitive science finds in the Information Processing Psychology (Newell and Simon, 1972) one important precursor. In the approach of Newell and Simon, a human agent is given a problem solving task, typically a logic exercise or the choice of moves in a chess game, asking him to think aloud, thus obtaining a verbal account of her mental processes while carrying out the task. Verbal reports are analyzed in order to identify the solution strategies adopted by the agent and the specific operations performed while carrying out the task. The analyzed verbal reports are then used to develop a program that simulates the behavior of the human agent. Subsequently, new problem solving tasks are given to both the program and the human agent, and verbal reports of the latter are compared with the execution traces of the simulative program to ascertain that the two systems use the same solution strategies. Finally, the program execution traces for new tasks are used for predicting the strategies and mental operations that the human agent will perform when given the same tasks.

Even though NLMs have been developed with engineering purposes only, namely for developing language processing systems, the early works of Caucheteux et al. (2023) and Kumar et al. (2023) show how they are being fruitfully applied to simulative AI as well.^3^ However, the way NLMs are used to predict and explain brain activations in the cortex puts significant methodological challenges for the epistemology of computer simulations and simulative AI.

One first main difference between the synthetic method in AI and the application of NLMs in neuroscience is that, as already noted, NLMs are not developed as simulative models of the brain. In other words, NLMs do not implement mechanisms that one hypotheses realize some cognitive function or capacity in a natural cognitive system. The aim of NLMs is not that of corroborating any such kind of hypotheses, as it happens with the simulative meethod in traditional AI. And nonetheless, NLMs are used to simulate the brain, that is, to obtain predictions of cortex activations.

The second, crucial, difference concerns the kind of simulative method that is being applied. The epistemology of computer simulation (Winsberg, 2022) is careful to notice that, beyond the simulation relation, two other central relations in the simulative method are those of *verification* and *validation*. Whereas verification is about ascertaining that the simulative computational system be a correct implementation of the mathematical model,^4^ validation is about evaluating the extent to which the mathematical model is a faithful representation of the target simulated system. For instance, Primiero (2020) states that a computational model and a target system can entertain a strong validation, when the computational model represent all and only the behaviors of the target system, a weak validation, when the computational model represent all but not only the behaviors of the target system, and an approximate validation when the computational model represent just some of the behaviors of the target system. As the studies of Caucheteux et al. (2022, 2023) clearly show, the neuroanatomy of the cortex is used to obtain hypotheses about the hierarchical layer organization of the Transformer. The hypotheses are tested by looking for new correlations between the depth of the Transformer layer activation and brain hierarchy level activations. In other words, the cortext is used as a model of the NLM. Since NLM are highly opaque, hypotheses about the inner organization of the ANN are obtained by looking at the brain. Once those hypotheses are tested, the NLM is used back to predict and explain brain activations.

Thus, from one hand, NLMs are used to provide simulative explanations of the brain in terms of model-based predictions of cortex activations. On the other hand, and relatively to the main aim of this paper, the brain itself is used to explain and simulate the NLM in what can be considered a sort of reciprocal or *co-simulation* (Angius et al., 2024). Both the brain language processing and the Transfomer language processing are opaque and poorly known processes and they are used to investigate and advance hypotheses about each other. The epistemological strategies herein collected as co-simulation can potentially reveal structural similarities between Transformers and the human brain, suggesting that this similarity may–at least in part–justify the Transformer's ability to handle natural language. Additional support comes from the scaling factor effect of the models. The number of model parameters still falls short of the estimated 10^14^ synapses in the neocortex, but it has emerged that structural similarity with the brain improves as the scale of the models increases. Antonello et al. (2023) found that structural similarity between brain and Transformer scales logarithmically with model size from 125M to 30B parameter models.

We add that co-simulation is currently the strategy pursued by researchers in comparing Transformers and the brain, but it is not the only option. An alternative could be interventionism, which we have seen in action within the context of mechanistic explanations (Section 5), and it is a fruitful methodology in neuroscientific research (Woodward, 2018). However, to our knowledge, there are no examples in the comparison between NLM and the brain.

## 7 Concluding remarks

In this article we have tried to answer the question: How is it possible for NLMs to show significant performance suggesting that they are intelligent and linguistically competent speakers? We have assumed that NLMs somehow pass the Turing test and that artificial semantics is not a mere chimera. However, we wonder how all this was possible. What has caught our attention in the argument is the empirical (*a posteriori*) possibility of artificial semantics and not its metaphysical possibility (*a priori*). How was it possible, given that it does work? It is worth noting that the impressive results demonstrated by NLMs did not come about in the expected way, namely by mimicking what the human body actually does when it exhibits the same cognitive abilities naturally. While there are interesting similarities between how NLMs work and some neuroscientific findings about language processing (see Section 6), overall the reason why remarkable results have been achieved in the language domain over the last five years is not inspired by the brain, as mentioned above. Rather, it is more elegant mathematics that is able to accomplish tasks that used to require equipment so complicated that they seemed overwhelming. Although there is no mystery about what a Transformer does, it must be admitted that there is a certain opacity in making sense of how Transformer produces individual cases of meaningful sentences and relevant answers. This opacity, combined with the generative mechanism that allows NLMs to produce new text each time, makes for a surprising sense of linguistic unpredictability. Sometimes we experience this feeling in the form of the Uncanny Valley (Mori, 1970)—that is, a phenomenon whereby entities that closely resemble humans yet exhibit subtle imperfections in appearance or behavior elicit cognitive dissonance, resulting in a unease or even discomfort—while at other times it seems as if we are dealing with an interlocutor “like me”.

Moreover, the way NLMs work does not seem to require the functional architectures that characterize cognitive science, or at least it is not necessary for such architectures to be represented in the way that has been common so far. Nevertheless, NLMs are typical creatures of cognitive science. They are computational constructs that generate the desired behavioral patterns by processing quantitatively represented information. The fact that this happens in sometimes unexpected ways has positive implications for the ecology of the communicative relationship between human interlocutors and NLMs. The feeling that we are dealing with an automaton often fades, and instead it seems as if we are dealing with a different kind of intelligence, as can be the case with strange individuals or other animals. NLMs have proven something we have basically always known: if we were to finally create an artificial intelligence, it would be different from natural intelligence. For example, since NLMs are trained on extremely vast corpora, they exhibit a cognitive style that reflects their advantage in information availability compared to a human. On the other hand, the world is not lacking in natural intelligence, and the advantage of artificial intelligence seems to lie precisely in its stylistic difference from human intelligence. So we can take advantage of the linguistic skills that NLMs perform effortlessly and that are tedious and boring for us. NLMs are still halfway between a prosthesis for humans and a real fellow. It is still uncertain where this adventure will lead, but by examining how all this has been possible so far, we can hope to gain useful information to discipline our imagination as to the outcome of what is happening.

## Data Availability

The original contributions presented in the study are included in the article/supplementary material, further inquiries can be directed to the corresponding author.

## Footnotes

^1^We are very grateful to one reviewer for having raised this important obiection.

^2^We are very grateful to one reviewer for having pointed out this question.

^3^It should be indeed recalled that AI has been historically characterized by two main research traditions, an engineering one concerning the development of artificial system showing intelligent behavior, and a simulative one using artificial intelligent systems to study natural cognitive systems (Russell and Norvig, 1995).

^4^Verification is used here in the proper sense it has in computer science: given a specification declaring the program requested functions, a computational system is correct with respect to a specification in case it fulfills the specification or, in other words, if it behaves as requested by the specification (Turner, 2018).
